# Inferences for current chronic graft-versus-host-disease free and relapse free survival

**DOI:** 10.1186/s12874-022-01771-x

**Published:** 2022-12-13

**Authors:** Xu Zhang, Scott R. Solomon, Connie Sizemore

**Affiliations:** 1 Center for Clinical and Translational Sciences, The University of Texas Health Science Center at Houston, Houston, TX, US; 2 The Blood and Marrow Transplant Program at Northside Hospital, Atlanta, GA, US

**Keywords:** Multistate, GRFS, Product integral, Kaplan-Meier estimator

## Abstract

This paper provides the methodologies of a new summary curve that measures the dynamic outcome following allogenic hematopoietic cell transplantation. This new summary curve computes the probabilities that a patient is alive in remission and free of severe-to-moderate chronic graft-versus-host disease (GVHD) over time. The probability is called Current chronic GVHD-free, Relapse-Free Survival (CGRFS). Based on a multistate model depicting the possible states that a patient may experience after transplant, CGRFS can be formulated as a linear combination of five survival functions. This method is known as the model-free approach. In this paper we provide the inferences of the model-free approach, including estimation of CGRFS, precision evaluation and comparison of CGRFS between two independent samples.

## Introduction

Several survival curves are often depicted to show the outcomes after allogenic hematopoietic cell transplantation. Among these curves, overall survival (OS) and disease-free survival (DFS) are most commonly used. Death and death/disease relapse are the endpoints for OS and DFS, respectively. Aiming at depicting the probability of survival in remission and free of comorbidity, Holtan et al. [1] proposed the GVHD-free, Relapse-Free Survival (GRFS). For GRFS, either death, relapse, grade 3-4 acute GVHD, or chronic GVHD requiring immunosuppression is viewed as a terminal failure event. GRFS is effective in measuring the short-term transplant outcome. In a study cohort of 907 patients, Holtan reported the 1-year GRFS to be 31%. The Center for International Blood and Marrow Transplant Research (CIBMTR) suggested 23% to be the reference 1-year GRFS in adult patients in trial studies. GRFS is very useful in trials evaluating different treatments since the target events can be observed within a relatively short time period.

GRFS is not suitable for assessing long-term post-transplant outcomes. First, acute GVHD occurred shortly after transplant. It is more appropriate to consider acute GVHD as a brief transit state. Second, chronic GVHD condition can be resolved in a large proportion of patients. There is no difference in quality of life between patients with resolved chronic GVHD and those never experiencing chronic GVHD. To better evaluate the long-term outcome, our group proposed the Current chronic GVHD-free, Relapse-Free Survival (CGRFS) function, defined as the probability that a patient is alive in remission and free of severe-to-moderate chronic GVHD after transplant at time *t* [2]. In CGRFS chronic GVHD is not a terminal event. A subject is removed from the risk set at the onset of chronic GVHD. When the chronic GVHD is resolved, the subject is again included in the risk set.

Composite endpoints such as DFS or GRFS are commonly used for summarizing outcomes in stem cell transplant studies. For a composite endpoint, only one health state is of study interest. CGRFS is not a composite endpoint since more than one health state are considered in CGRFS. The dynamic nature of CGRFS makes it similar to the current leukemia-free survival (CLFS) proposed by Klein, Szydlo et al. [3]. CLFS was advocated as a better summary curve measuring effectiveness of donor lymphocyte infusion (DLI) post transplant [4]. Post-transplant relapse was treated by DLI and many relapsed patients achieved the second remission. For CLFS, relapse in the initial remission is not a terminal event. Both initial and second remission states are considered in CLFS.

A multistate model should be constructed for a dynamic endpoint [5]. The survival associated with a dynamic endpoint involves the transition probabilities from the initial state to other states. The conventional method for estimating a transition probability is the product-limit estimator [6]. An alternative method for estimating a transition probability was suggested by Pepe [7] by using the difference of two survival functions. For CLFS, Klein, Szydlo et al. [3] employed the conventional product-limit method to estimate the transition probability in CLFS. In another work by Klein, Keiding et al. [8], inspired by Pepe’s idea, they proposed the model-free approach to formulate CLFS as a linear combination of three survival functions, and use the Kaplan-Meier estimators to estimate the survival functions. For CGRFS, both conventional product-limit method and the model-free approach can be utilized. The model-free approach has a great advantage because it is much simpler in formulation and computation. In our clinical paper, we suggested the model-free approach and presented the estimation result of a real data set [2]. In recent years other dynamic endpoints have been advocated for stem cell transplant studies [9, 10]. The current survival functions were defined for these dynamic endpoints based on specific multistate models, and the model-free approach was utilized to estimate the proposed current survival functions.

CGRFS has been recognized as a useful addition to the existing survival functions [11–13] and considered as suitable implication of transplant success [14]. Our clinical paper did not include interval estimation, which is critical for assessing precision of CGRFS estimates. Also the clinical paper lacked details about two-sample comparison. In this paper we present the detailed inferences. In Section 2 we give definition of CGRFS, together with point estimation, precision evaluation and two-sample comparison. In Section 3 we present the analytical results of the data of 422 patients, including the CGRFS curve, the estimated transition probabilities of different health states, and two-sample comparison result. In addition, we explain how to compute probabilities of all states, and suggest other practically meaningful functions. A discussion of CGRFS is given in Section 4.

## Methods

### Estimation of CGRFS

For CGRFS, onset and resolution of chronic GVHD need to be clearly determined. Onset of chronic GVHD event was defined as moderate-to-severe chronic GVHD based on the NIH criteria [15] at the time of the most recent assessment. GVHD evaluation was prospectively performed by a single practitioner within the program. Resolution of chronic GVHD was determined if symptoms became quiescent and systemic immunosuppression discontinued.

**Fig. 1:**
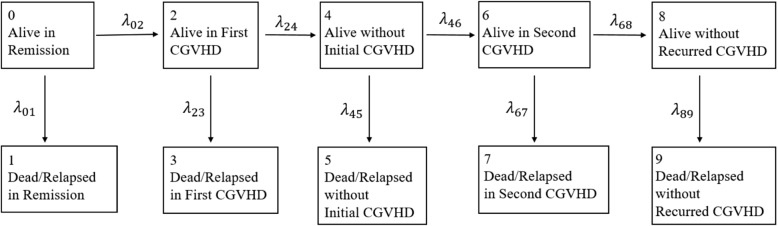
Possible transitions in the multistate model for CGRFS

We constructed a multi-state model to depict the disease progression after transplantation. Two episodes of chronic GVHD were incorporated in the model (Fig. 1). The model contains the following states,

State 0: Alive in initial remission state without experiencing chronic GVHD

State 1: Dead or relapsed before first chronic GVHD

State 2: Alive with first chronic GVHD

State 3: Dead or relapsed in first chronic GVHD

State 4: Alive without first chronic GVHD

State 5: Dead or relapsed in recovery from first chronic GVHD

State 6: Alive with second chronic GVHD

State 7: Dead or relapsed in second chronic GVHD

State 8: Alive without second chronic GVHD

State 9: Dead or relapsed in recovery from second chronic GVHD

CGRFS is the probability that one stays in state 0 or 4 or 8 at time *t*. Let $P_{kl}(s,t)$ be the transition probability from state *k* to *l* in time interval [*s*, *t*]. CGRFS, denoted by *C*(*t*), is defined as the sum of three transition probabilities, $C(t)=P_{00}(0,t)+P_{04}(0,t)+P_{08}(0,t)$. An intensity matrix can be constructed based on the transition intensities, $\lambda _{01},\cdots ,\lambda _{89}$ (Fig. 1). The conventional method of estimating $P_{00}(0,t), P_{04}(0,t)$ and $P_{08}(0,t)$ is to consider the product integral of the intensity matrix [6].

The product-integral method is computationally expensive. The issue is most severe for $P_{08}(0,t)$ because a subject has to experience three states before reaching state 8, alive without the second chronic GVHD. Following Pepe [7] and Klein, Keiding et al. [8], we developed the model-free approach for CGRFS [2]. We formulated CGRFS as a linear combination of five survival functions, $S_1(t),\cdots ,S_5(t)$, pertaining to five composite endpoints. These five composite endpoints are explained as follows,

$S_1(t)$: First occurrence of first chronic GVHD or death/relapse,

$S_2(t)$: First occurrence of second chronic GVHD or death/relapse,

$S_3(t)$: First occurrence of resolution of first chronic GVHD or death/relapse,

$S_4(t)$: Death/relapse at any time,

$S_5(t)$: First occurrence of resolution of second chronic GVHD or death/relapse.

Let $T_{k}$ be the time to the *k*th composite endpoint and $S_k(t)=Pr(T_k > t)$ ($k=1,\cdots ,5$). Note that $S_1(t)$ coincides with $P_{00}(0,t)$. The composite endpoint for $T_2$ is the first occurrence of second chronic GVHD or death/relapse. For $T_2$, the death/relapse event could only be death/relapse in remission, in first chronic GVHD or in resolved first chronic GVHD. A patient without experiencing this composite endpoint stays in state 0, 2 or 4 (see Fig. 1). Using transition probabilities, $S_2(t)=\mathrm {Pr}(T_2>t)=P_{00}(0,t)+P_{02}(0,t)+P_{04}(0,t)$. Similarly, the composite endpoint for $T_3$ is the first occurrence of resolution of first chronic GVHD or death/relapse. Without experiencing this composite event, one stays in state 0 or 2. That is, $S_3(t)=\mathrm {Pr}(T_3>t)=P_{00}(0,t)+P_{02}(0,t)$. Consequently, $S_2(t)-S_3(t)$ yields $P_{04}(0,t)$. In addition, based on the composite endpoints for $T_4$ and $T_5$, $S_4(t)$ is the probabilities of staying in state 0, 2, 4, 6 or 8 while $S_5(t)$ is the probability of staying in state 0, 2, 4 or 6. We can get that $P_{08}(0,t)=S_4(t)-S_5(t)$. In summary, CGRFS is a linear combination of these five survival functions, $C(t)=S_1(t)+S_2(t)-S_3(t)+S_4(t)-S_5(t)$. Among these survival functions, $S_1(t)$ and $S_4(t)$ are practically meaningful as they are the relapse-free, chronic GVHD free survival and DFS, respectively. Other survival functions are not interpretable in real life but introduced here to find probability of being in one state. All these five survival functions can be estimated by the Kaplan-Meier method.

To introduce inferences for CGRFS, we used the counting process notations. Let *Z* be the censoring time. Suppose that data of *n* patients are collected. The sample can be summarized as $(X_{ik}, \Delta _{ik})\ (i=1,\cdots ,n;\ k=1,\cdots ,5)$, where $X_{ik}=\min (T_{ik}, Z_i),\ \Delta _{ik}=I(T_{ik}\leq Z_i)$ and $I(\bullet )$ is an indicator function which takes value 1 if the event happens, and 0 otherwise. $N_{ik}(t)$ indicates whether the *i*th patient experiences the *k*th composite endpoint at or prior to *t*, that is, $N_{ik}(t)=I(X_{ik}\leq t, \Delta _{ik}=1)$ and let $\bar{N}_k(t)=\sum _{i=1}^n N_{ik}(t)$. Also we defined the risk sets related to the five composite endpoints. Let $Y_{ik}(t)=I(X_{ik}\geq t)$ and $\bar{Y}_k=\sum _{i=1}^n Y_{ik}(t)$. So $\bar{Y}_1(t)$ is number of patients alive in remission without experiencing CGVHD, i.e., in state 0 at time *t*, and

$\bar{Y}_2(t)$: Number of patients in states 0, 2, 4 at time *t*

$\bar{Y}_3(t)$: Number of patients in states 0, 2 at time *t*

$\bar{Y}_4(t)$: Number of patients in states 0, 2, 4, 6, 8 at time *t*

$\bar{Y}_5(t)$: Number of patients in states 0, 2, 4, 6 at time *t*

Using the counting process notations, the Kaplan-Meier estimator of $S_k(t)$ is given by

$$\begin{aligned} \widehat{S}_k(t)=\prod\limits_{u\geq 0}^t \left[ 1-\frac{d\bar{N}_k(u)}{\bar{Y}_k(u) } \right] , \quad k=1,\cdots , 5. \end{aligned}$$

CGRFS, as a linear combination of five survival functions, can be subsequently estimated by $\widehat{C}(t)=\widehat{S}_1(t)+\widehat{S}_2(t)-\widehat{S}_3(t)+\widehat{S}_4(t)-\widehat{S}_5(t)$.

The hazard function of $T_k$ is defined as $\lambda _k(t)=\lim _{\Delta t\rightarrow 0}\mathrm {Pr}(t\leq T_{k} < t+\Delta t | T_k\geq t)/\Delta t$. Then we have the martingales

$$\begin{aligned} M_{ik}(t)=N_{ik}(t)- \int _0^t Y_{ik}(u) \lambda _k(u)du, \quad i=1,\cdots ,n;\ k=1,\cdots ,5. \end{aligned}$$

Define $y_k(t)=n^{-1}\lim _{n\rightarrow \infty } \bar{Y}_k(t), \forall k=1,\cdots ,5$. Based on the facts in Andersen et al. [6] and Pepe [7], for large samples,

$$\begin{aligned} \sqrt{n}\left[ \widehat{C}(t)-C(t) \right] \approx \frac{1}{\sqrt{n}}\sum \limits _{i=1}^n W_i(t), \end{aligned}$$

where

$$\begin{aligned} W_i(t)= & {} -S_1(t)\int _0^t \frac{dM_{i1}(u)}{y_1(u)} -S_2(t)\int _0^t \frac{dM_{i2}(u)}{y_2(u)}+S_3(t)\int _0^t\frac{dM_{i3}(u)}{y_3(u)}\\&-S_t(t)\int _0^t \frac{dM_{i4}(u)}{y_4(u)}+S_5(t)\int _0^t\frac{dM_{i5}(u)}{y_5(u)} \end{aligned}$$

Based on the martingale central limit theorem, $\forall t$, $\sqrt{n}\left[ \widehat{C}(t)-C(t) \right]$ converges to a mean-zero normal distribution with variance $\sigma (t)^2=E[ W_i(t)^2]$. It should be noted that the martingale processes, $M_{i1}(t), \cdots , M_{i5}(t)$, are not orthogonal because by definition the event counting processes may involve the same events. Consequently the covariance should be considered if one wishes to consider the martingale variation process. Here we alternatively consider a moment estimator of the variance, $\widehat{\sigma }(t)^2=n^{-1}\sum _{i=1}^n [ \widehat{W}_i(t)^2]$, where

$$\begin{aligned} \widehat{W}_i(t)= & {} n\left[ -\widehat{S}_1(t)\int _0^t \frac{\widehat{M}_{i1}(u)}{\bar{Y}_1(u)} -\widehat{S}_2(t)\int _0^t \frac{\widehat{M}_{i2}(u)}{\bar{Y}_2(u)}+\widehat{S}_3(t)\int _0^t\frac{\widehat{M}_{i3}(u)}{\bar{Y}_3(u)} \right. \\&\left. -\widehat{S}_t(t)\frac{\widehat{M}_{i4}(u)}{\bar{Y}_4(u)}+\widehat{S}_5(t)\int _0^t \frac{\widehat{M}_{i5}(u)}{\bar{Y}_5(u)} \right] \end{aligned}$$

and

$$\begin{aligned} \widehat{M}_{ik}(t)=N_{ik}(t)- \int _0^t \frac{dN_{ik}(u)}{\bar{Y}_k(u)} . \end{aligned}$$

The above moment variance estimator is similar to the variance estimation method provided by Klein, Keiding et al. [8] for current LFS. A linear $(1-\alpha )100\%$ confidence interval for CGRFS can be calculated by $\widehat{C}\pm n^{-1/2}z_{1-\alpha /2}\ \widehat{\sigma }(t)$. Log-log transformation is routinely used for interval estimation of a survival probability. A $(1-\alpha )100\%$ log-log transformed confidence interval for *C*(*t*) is given by

1 $$\begin{aligned} \left[ \widehat{C}(t)^{1/\theta },\ \widehat{C}(t)^\theta \right] \ \ \mathrm {with}\ \ \theta =\exp \left( \frac{n^{-1/2}z_{1-\alpha /2}\ \widehat{\sigma }(t)}{\widehat{C}(t)\ln \left[ \widehat{C}(t)\right] }\right) . \end{aligned}$$

### A confidence band for CGRFS

In survival analysis the simulation approach has been commonly used to find the confidence band of a survival function [16, 17]. $\forall i,k$, let $G_{ik}(t)$ be a standard normal random variate. The martingale process $\int _0^t dM_{ik}(u)$ has the same distribution as $\int _0^t G_{ik}(u)dN_{ik}(u)$. Based on this knowledge, we consider the process $\widetilde{W}(t)$ that

2 $$\begin{aligned} \widetilde{W}(t)= & {} \frac{1}{\sqrt{n}}\sum \limits _{i=1}^n \left[ -S_1(t)\int _0^t \frac{G_{i1}(u)dN_{i1}(u)}{y_1(u)} -S_2(t)\int _0^t \frac{G_{i2}(u)dN_{i2}(u)}{y_2(u)}\right. \nonumber \\&\left. +\ S_3(t)\int _0^t\frac{G_{i3}(u)dN_{i3}(u)}{y_3(u)} -S_t(t)\int _0^t \frac{G_{i4}(u)dN_{i4}(u)}{y_4(u)}+S_5(t)\int _0^t\frac{G_{i5}(u)dN_{i5}(u)}{y_5(u)} \right] . \end{aligned}$$

Let $Q(t)=\left| \sqrt{n}\left\{ \widehat{C}(t) -C(t)\right\} \right|$, then *Q*(*t*) can be approximated by $\widetilde{W}(t)$. To construct a band for *C*(*t*) in an interval $[t_1,t_2]$, one needs to find the critical value such that

$$\begin{aligned} \mathrm {Pr}\left( {\underset{t\in [t_1,t_2]}{\sup}} \left| Q(t) /\sigma (t)\right| >q_\alpha \right) =\alpha . \end{aligned}$$

To obtain a realized process, one can generate standard normal random variates for $G_{ik}(t),\forall i, k$. Also plug in the Kaplan-Meier estimators for survival probabilities and $Y_k(t)/n$ to replace $y_k(t)$,

3 $$\begin{aligned} \widehat{\widetilde{W}}(t)= & {} \sqrt{n}\sum \limits _{i=1}^n \left[ -\widehat{S}_1(t)\int _0^t \frac{G_{i1}(u)dN_{i1}(u)}{\bar{Y}_1(u)} -\widehat{S}_2(t)\int _0^t \frac{G_{i2}(u)dN_{i2}(u)}{\bar{Y}_2(u)}\right. \nonumber \\&\left. +\ \widehat{S}_3(t)\int _0^t\frac{G_{i3}(u)dN_{i3}(u)}{\bar{Y}_3(u)} -\widehat{S}_t(t)\int _0^t \frac{G_{i4}(u)dN_{i4}(u)}{\bar{Y}_4(u)}+\widehat{S}_5(t)\int _0^t\frac{G_{i5}(u)dN_{i5}(u)}{\bar{Y}_5(u)} \right] . \end{aligned}$$

Given *B* realized processes, let $\widehat{\widetilde{W}}_b(t)$ denote the *b*th realized process. The critical value is obtained by finding the $(1-\alpha )100th$ percentile of the supremum values, which is given by

$$\begin{aligned} B^{-1}\sum\limits_{b=1}^B\mathcal {I}\left( {\underset{t\in [t_1,t_2]}{\sup}} \left| \widehat{\widetilde{W}}_b (t)/\widehat{\sigma }(t)\right| > \tilde{q}_\alpha \right) =\alpha , \end{aligned}$$

where $\mathcal {I}(\bullet )$ is the indicator function. A confidence band for *C*(*t*) is given by $\widehat{C}(t)\pm n^{-1/2}\tilde{q}_\alpha \widehat{\sigma }(t), \forall t \in [t_1,t_2]$.

### A confidence band for differences in CGRFS between two independent samples

The method described in Section 2.2 can be extended to construct a confidence band for differences in CGRFS between two independent samples. Such a band could tell in what time range that CGRFS’s of two groups differ. This type of band is related to the hypotheses $H_0: C_1(t)-C_2(t)=0$ versus $H_a: C_1(t)-C_2(t)\ne 0, \forall t \in [t_1,t_2]$, where $C_i(t)$ is CGRFS for sample *i*. A supremum test for the hypotheses can be developed.

Let $\widehat{C}_1(t)$ and $\widehat{C}_2(t)$ be the estimated CGRFS of samples 1 and 2, respectively. Let the processes described in Eq. (2) for samples 1 and 2 denoted by $\widetilde{W}^{(1)}(t)$ and $\widetilde{W}^{(2)}(t)$, respectively. Under the null hypothesis, asymptotically $\sqrt{n}\left\{ C_1(t)-C_2(t)\right\}$ has the same distribution as $\widetilde{W}^{(1)}(t)- \widetilde{W}^{(2)}(t)$. To obtain a standardized realized process, we need to estimate the standard error for $\sqrt{n}\left\{ C_1(t)-C_2(t)\right\}$, $\widehat{SE}(t)=\sqrt{\widehat{\sigma }_1(t)^2+\widehat{\sigma }_2(t)^2}$, where $\widehat{\sigma }_i(t)^2 ( i=1,2)$ is the estimated variances for sample *i*. A standardized realized process is defined as $\widehat{U}(t)=\left\{ \widehat{\widetilde{W}}^{(1)}(t)- \widehat{\widetilde{W}}^{(2)}(t)\right\} / \widehat{SE}(t)$. We can generate *B* realized processes

$$\begin{aligned} \widehat{U}_b(t)=\left\{ \widehat{\widetilde{W}}^{(1)}_b(t)- \widehat{\widetilde{W}}^{(2)}_b(t)\right\} /\widehat{SE}(t),\ b=1,\cdots ,B. \end{aligned}$$

The critical value for the band can be obtained by finding the $(1-\alpha)100th$ percentile among *B* supremum values,

$$\begin{aligned} B^{-1}\sum\limits_{b=1}^B\mathcal {I}\left( {\underset{t\in [t_1,t_2]}{\sup}} \left| \widehat{U}_b (t)\right| > \tilde{g}_\alpha \right) =\alpha . \end{aligned}$$

The $(1-\alpha )100\%$ confidence band for $C_1(t)-C_2(t)$ is given by $\widehat{C}_1(t)-\widehat{C}_2(t) \pm n^{-1/2}\tilde{g}_\alpha \widehat{SE}(t)$.

For the supremum test, we evaluate the supremum for the sample data,

$$\begin{aligned} \widehat{K}={\underset{t\in [t_1,t_2]}{\sup}}\left| \sqrt{n}\left\{ \widehat{C}_1(t)-\widehat{C}_2(t)\right\} /\widehat{SE}(t)\right| . \end{aligned}$$

The test *p*-value is to the probability of observing $\widehat{K}$ or more extreme values in the sampling distribution of $\sup _{t\in [t_1,t_2]}\left| \widehat{U}(t)\right|$. The *p*-value can be obtained by finding the proportion of the supremum values higher or equal to $\widehat{K}$,

$$\begin{aligned} p=B^{-1} \sum\limits_{b=1}^n\mathcal {I} \left( {\underset{t\in [t_1,t_2]}{\sup}}\left| \widehat{U}_b(t)\right| \geq \widehat{K}\right) . \end{aligned}$$

In a supremum test, one should determine the time interval $[t_1,t_2]$ in which survival functions of two samples are compared. If the goal is to compare survival functions over the entire study period, one may set $t_1=\max \left( t_{\mathrm {min}}^{(1)}, t_{\mathrm {min}}^{(2)}\right)$ where $t_{\mathrm {min}}^{(1)}$ and $t_{\mathrm {min}}^{(2)}$ are the smallest event times in samples 1 and 2, respectively. If a large number of subjects remain under study even after the largest event time, one may set $t_2$ to be the largest event time in two samples.

## The real life example

The study cohort consisted of 422 patients receiving an allogeneic transplant at a single institution in 2010 to 2015. The median age was 44 years (range 18 - 77). 56% of patients were male. Matched related, matched unrelated and haploidentical donors were used in 125, 165 and 132 patients, respectively. The majority had low or intermediate disease risk index (DRI) (N=291, 69%). About half had hematopoietic cell transplant comorbidity index (HCT-CI) 3 or higher (N=194, 46%). Among 264 survivors the median follow-up time was 36 months (range 11-78 months).

**Fig. 2:**
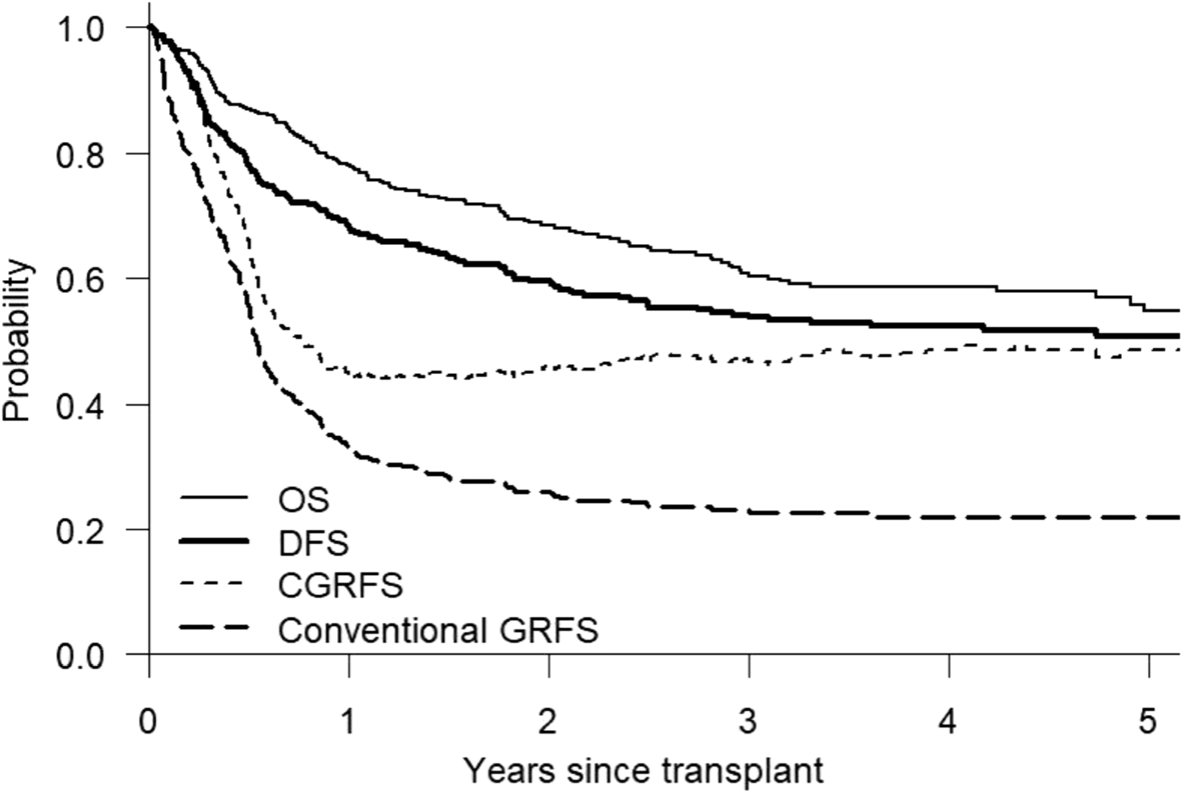
OS, CGRFS and conventional GRFS curves

We depicted four survival curves in Fig. 2. The 1-year OS, DFS, conventional GRFS and CGRFS (Table 1) were 0.78 (95% CI 0.74-0.82), 0.68 (95% CI 0.64-0.72), 0.33 (95% CI 0.29-0.38) and 0.45 (95% CI 0.40-0.50), respectively. The 1-year GRFS of this cohort was comparable to the rate reported by Holtan et al. [1] based on a cohort of 907 patients. As shown in Fig. 2, the GRFS curve dropped rapidly within 1 year, followed by a slow decrease in 1 to 3 years, and then the curve became flattened after 3 years. Since majority of events for GRFS occurred within one year, this function is only good for assessing the short-term outcome. At 1 year, the CGRFS estimate was about 0.12 higher than the GRFS estimate and the difference became greater afterwards. Two reasons explain the difference. First, definition of CGRFS does not involve acute GVHD event. Second, a good proportion of chronic GVHD conditions were resolved. At 3 years, OS, DFS, GRFS and CGRFS estimates were 0.61 (95% CI 0.55-0.66), 0.54 (95% CI 0.49-0.59), 0.23 (95% CI 0.18-0.27) and 0.47 (95% CI 0.42-0.52), respectively. CGRFS and DFS estimates were very different at 1 year but became similar at 3 years, indicating that a large proportion of chronic GVHD conditions were resolved.

**Table 1:** Estimates of survival probabilities and 95% confidence intervals at different time points

	OS		DFS		GRFS		CGRFS	
Time	Estimate	95% CI	Estimate	95% CI	Estimate	95%	Estimate	95%
1 year	0.78	(0.74, 0.82)	0.68	(0.64, 0.72)	0.33	(0.29, 0.38)	0.45	(0.40, 0.50)
2 years	0.69	(0.64, 0.73)	0.60	(0.55, 0.64)	0.26	(0.22, 0.30)	0.46	(0.41, 0.51)
3 years	0.61	(0.55, 0.66)	0.54	(0.49, 0.59)	0.23	(0.18, 0.27)	0.47	(0.42, 0.52)
4 years	0.59	(0.53, 0.64)	0.52	(0.47, 0.57)	0.22	(0.18, 0.26)	0.49	(0.43, 0.54)

**Fig. 3:**
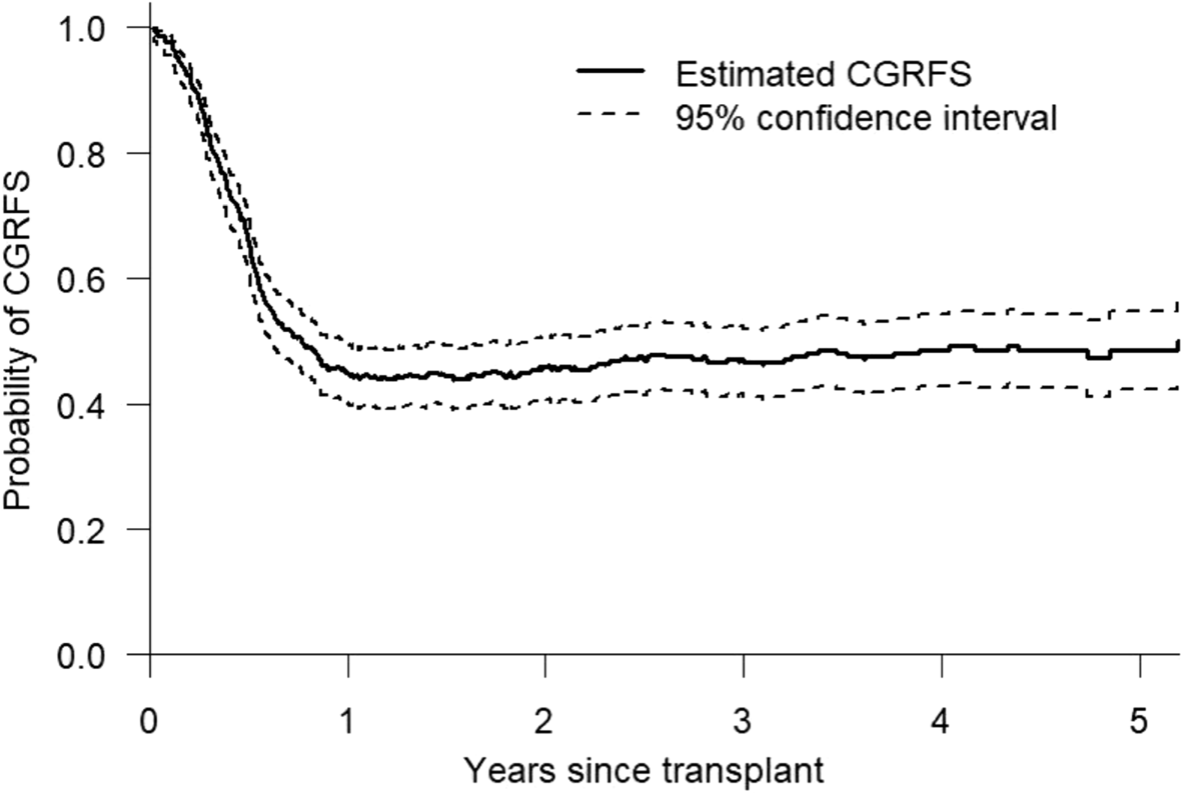
The CGRFS curve together with the 95% confidence interval

The CGRFS curve together with its 95% confidence interval were depicted in Fig. 3. The log-log transformation was employed for interval estimation. The confidence interval was calculated by the formula given in Eq. (1). We also evaluated the confidence interval for DFS (not shown in the paper). The confidence interval for CGRFS is narrower than that of DFS within 1 year but the precision levels for CGRFS and DFS become comparable afterwards.

**Fig. 4:**
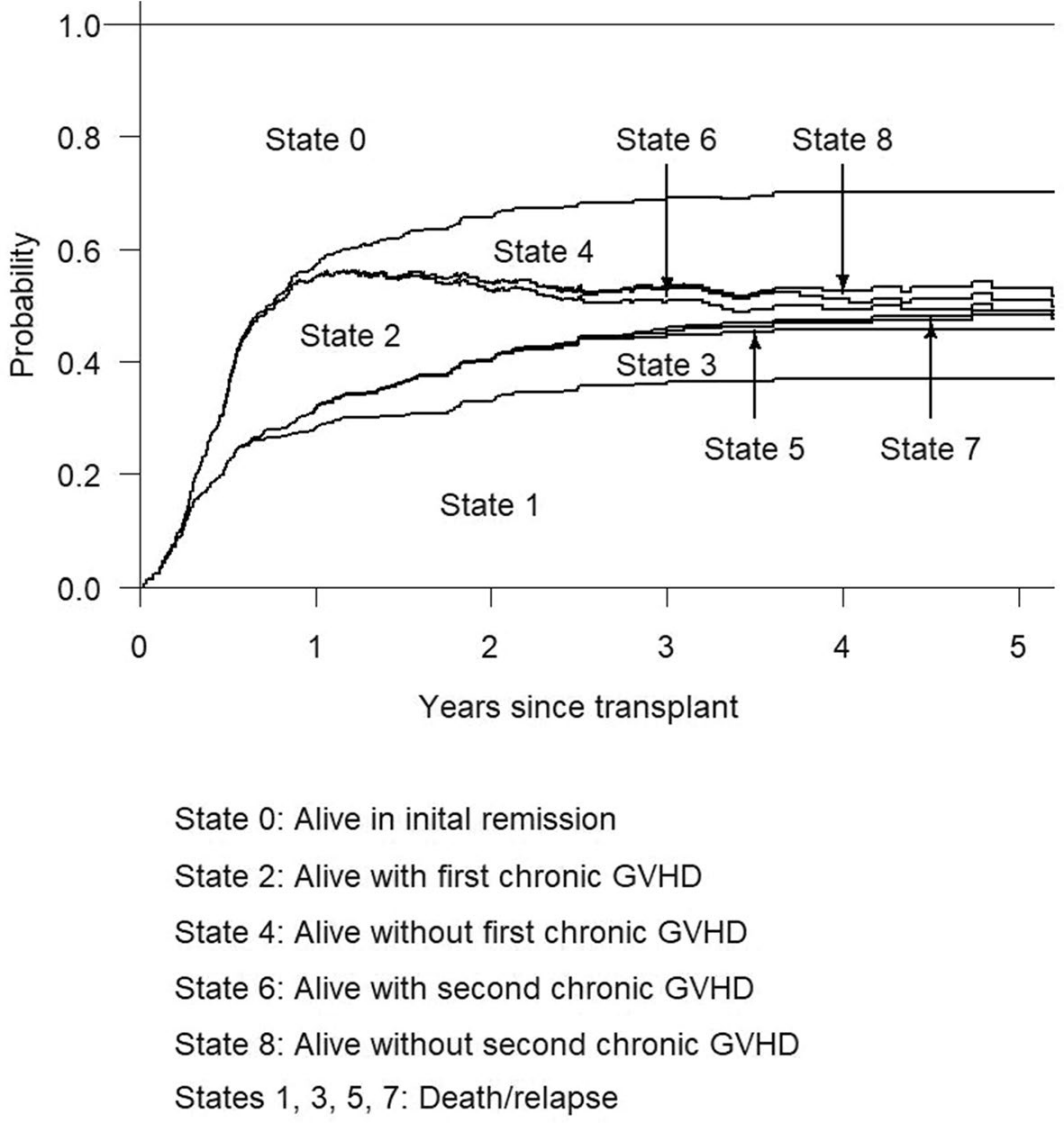
Estimated probabilities that a patient is in different states after transplant

Though we focused on the probabilities of being in states 0, 4 and 8 (alive free of chronic GVHD) in Fig. 1, it is not challenging to find the probabilities of other states. According to the composite endpoints and survival probabilities explained in Section 2.1, we can identify that $S_3(t)-S_1(t)$ and $S_5(t)-S_2(t)$ yield the probabilities of staying in state 2 and state 6, respectively. Note that states 2 and 6 relate to survival under chronic GVHD condition. Regarding probabilities of state 1, we can see that death/relapse in remission and onset of first chronic GVHD are two competing risks. Therefore, the probability of state 1 is the cumulative incidence function in the competing risks context and can be estimated by the Aalen-Johansen estimator. Probabilities of other death/relapse states can all be recognized as cumulative incidence functions under competing risks settings. In summary, for $i\in (1,3,5,7,9), \mathrm {Pr}(\mathrm {State}\ i)=P_{0i}(0,t)=\int _0^t P_{0(i-1)}(0,u-)\lambda _{(i-1)i} (u)du$. These functions can be estimated by the Aalen-Johansen estimator. In Fig. 4 we depicted the probabilities of being in states 1 to 8. We can see from this figure that very small proportions of patients stayed in states 5 and 7, indicating that patients with resolved chronic GVHD had low chance of experiencing failure events. Note that no one died while alive with resolved second chronic GVHD. Therefore, zero percent of patients stayed in state 9.

As shown in Fig. 4, 28% of patients had entered state 2 (first chronic GVHD) within one year. At 1 year, 23%, 3% and 2% of patients were staying in states 2, 3 (death/relapse in first chronic GVHD) and 4 (resolution of first chronic GVHD), respectively. These numbers indicate that a high proportion of patients developed chronic GVHD within one year of transplantation. In some patients, the chronic GVHD condition was resolved very soon as 2% became GVHD free by 1 year, while 3% died or relapsed by 1 year. As time went by, relative more patients resolved their GVHD condition rather than experienced failure event. At 2 years, 32% patients had developed initial chronic GVHD and entered state 2. Among them, 7% died or relapsed while 13% resolved chronic GVHD condition and transited to state 4. Only 12% were alive with GVHD condition. At 2 years, in patients with resolved GVHD, a small proportion of them (1.5%) experienced second episode of chronic GVHD, while the majority (11.5%) remained relapse-free and GVHD-free.

**Fig. 5:**
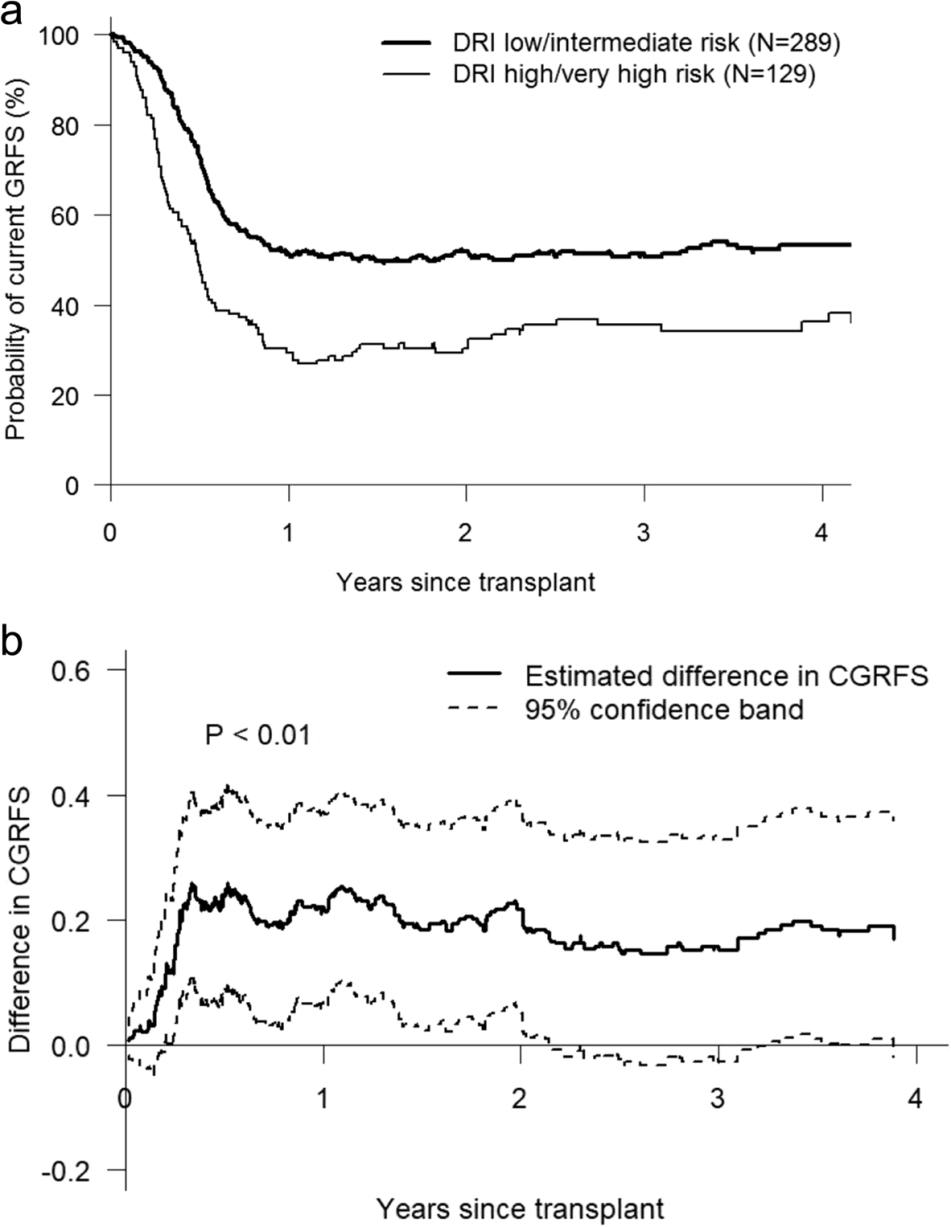
**a** CGRFS curves in DRI subgroups. **b** The 95% confidence band for difference in CGRFS between DRI subgroups

Association of demographics and clinical characteristics with CGRFS was evaluated by the supremum test described in Section 2.3. We chose to conduct the supremum test in the time interval $[t^*,4]$ years, where $t^*$ is the larger value between two smallest event times of two samples. Only a few CGRFS events occurred after 4 years. Therefore we truncated at 4 years to avoid the high variability at the tails of CGRFS curves. We evaluated the following factors: age (<55, $\ge$55), gender, Dana-Farber risk index (DRI) (low/intermediate, high/very high), HCT-CI (0-2, $\ge$3), donor type, stem cell source (bone marrow, PBSC), diagnosis, conditioning intensity, CMV status, and year of transplantation (2010-2012, 2013-2015). Based on the supremum test results, only DRI had significant effect on CGRFS ($P<0.01$). Compared to the high or very high risk in DRI, patients with low or intermediate risk had significantly higher chance to stay in leukemia and chronic GVHD free status (Fig. 5).

Other outcomes can be generated from the multistate model in Fig. 1. For example, Pepe [7] mentioned that the prevalence of chronic GVHD in leukemia-free patients provides a measure of quality of life. Based on the states in Fig. 1, this prevalence is given by

4 $$\begin{aligned} \frac{\mathrm {Pr}(\mathrm {State}\ 2)+\mathrm {Pr}(\mathrm {State}\ 6)}{\mathrm {Pr}(\mathrm {DFS})}. \end{aligned}$$

As another example, it is interesting to examine whether the chance of failure would be higher when a chronic GVHD is resolved in a patient compared to one staying in the initial remission state. To answer this question and given that we are interested in recovery from the first chronic GVHD only, we can consider the following function,

5 $$\begin{aligned} \frac{\mathrm {Pr}(\mathrm {State}\ 5)}{\mathrm {Pr}(\mathrm {State}\ 4)} - \frac{\mathrm {Pr} (\mathrm {State}\ 1)}{\mathrm {Pr} (\mathrm {State}\ 0) }. \end{aligned}$$

Based on our clarification on the probabilities of all states given in this section, it is straightforward to estimate the functions in Eqs. 4 and 5. The bootstrap method can be used for interval estimation.

## Discussion

Pepe [7] initially discussed the model-free approach for estimating probability of chronic GVHD. CGRFS was introduced to accommodate a more general context including two episodes of chronic GVHD. It reflects both onset and resolution of initial and recurred chronic GVHD. A CGRFS curve is a useful supplement to the conventional OS, DFS and GRFS curves. For DFS, a patient is alive without relapse but may still suffer from chronic GVHD. Different from DFS, CGRFS shows the probability of staying in a better health status and is a meaningful measure of good quality of life. In the example, we explained how to estimate the probabilities of all states. Based on these results, we will be able to find the probabilities of survival with chronic GVHD (sum of probabilities of states 2 and 6). Suppose that we consider CGRFS as the perfect health condition and assign the utility value 1. If a utility for survival with chronic GVHD is provided, the quality-adjusted lifetime can be evaluated. This quantity will be another tool for outcome assessment.

In this paper we presented the inferences for CGRFS. Using the model-free approach, CGRFS can be conveniently estimated by a linear combination of Kaplan-Meier estimators of five survival functions. Computation can be done by invoking the build-in functions in statistical software and performing basic data manipulation. More specifically, one can use a build-in function, such as the LIFETEST procedure in SAS, to compute Kaplan-Meier estimates of relevant survival functions. The next step is to merge the survival probability estimates by time, and then evaluate the linear combination. 

Suppose that there exists only one episode of chronic GVHD. Such a setting is described by states 0 to 5 only, which cover the states related to the first chronic GVHD, and CGRFS reduces to $S_0(t)+S_3(t)-S_2(t)$. The inferential methods provided in Section 2 can be simplified by removing terms related to $S_4(t)$ and $S_5(t)$.

Relapse in CGRFS is treated as a terminal event. If relapse would rather be considered as a curable condition, only minor changes are needed for the underlying multistate model. First, death becomes the only terminal event in states 1, 3, 5, 7 and 9 (states of the terminal event). Second, occurrence of either chronic GVHD or relapse triggers entrance to state 2 and state 6 (states of the diseases). Third, one in state 2 transits to state 4, as well as transition from state 6 to 8, where states 6 and 8 indicate disease resolutions, only if the person is free of chronic GVHD and relapse. Estimation methods discussed in the paper are still applicable for CGFRS based on such a multistate model.

For studies focus on post-GVHD performances, e.g., a study to evaluate efficacy of different treatments for chronic GVHD, the multistate model can be revised by removing states 0 and 1 (initial remission and death/relapse in remission). Under this reduced multistate model, the time origin becomes onset of chronic GVHD. Sum of probabilities of states 4 and 8 (survival without chronic GVHD) can be used as the function for outcome assessment. One can follow the methods presented in Section 2 to develop relevant inferences.

## Data Availability

A mock data set and a SAS program for estimating CGRFS are available from the corresponding author on request.
